# Immunotherapies for hepatocellular carcinoma

**DOI:** 10.1002/cam4.4468

**Published:** 2021-12-24

**Authors:** Justin K. H. Liu, Andrew F. Irvine, Rebecca L. Jones, Adel Samson

**Affiliations:** ^1^ Leeds Institute of Medical Research at St James's (LIMR) School of Medicine Faculty of Medicine and Health University of Leeds St James's University Hospital Leeds UK; ^2^ Leeds Liver Unit St James's University Hospital Leeds Teaching Hospitals NHS Trust Leeds UK

**Keywords:** adoptive cell therapy, cancer immunotherapy, dendritic cell therapy, hepatocellular carcinoma, immune checkpoint inhibitors, oncolytic viruses

## Abstract

Cases of hepatocellular carcinoma (HCC) are rapidly rising. This is particularly the case in the Western world, as a result of increasing rates of chronic liver disease, secondary to lifestyle‐associated risk factors and the lack of an established screening programme for the general population. Traditionally, radical/curative treatment options for HCC, including liver transplantation and surgical resection are reserved for the minority of patients, presenting with an early stage cancer. For patients with advanced disease, Sorafenib and Lenvatinib were, until recently, the only licensed systemic treatments, and provided only limited survival benefits at the cost of a multitude of potential side effects. Recent scientific advances in the field of cancer immunotherapy have renewed significant interest in advanced HCC, in order to fulfil this apparent area of unmet clinical need. This has led to the success and recent regulatory approval of an Atezolizumab/Bevacizumab combination for the first‐line treatment of advanced HCC following results from the IMbrave150 clinical trial in 2019, with further immune checkpoint inhibitors currently undergoing testing in advanced clinical trials. Furthermore, other cancer immunotherapies, including chimeric antigen receptor T‐cells, dendritic cell vaccines and oncolytic viruses are also in early stage clinical trials, for the treatment of advanced HCC. This review will summarise the major approaches that have been and are currently in development for the systemic treatment of advanced HCC, their advantages, drawbacks, and predictions of where this revolutionary treatment field will continue to travel for the foreseeable future.

## Graphical abstract

Cancer immunotherapies are emerging as one of the main faces in the new era of cancer treatment and personalised medicine. The cancer immunotherapy pharmaceutical industry is now worth billions of dollars and much research is now focused on harnessing their huge potential in treating a wide range of diseases. In this review, we discuss the different types of cancer immunotherapies currently licensed and in development for the treatment of hepatocellular carcinoma, their drawbacks and limitations, and future innovations that are currently being researched in order to maximise their effectiveness for use in clinical practice.

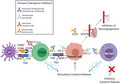

## 1 INTRODUCTION

Hepatocellular carcinoma (HCC) is the most common primary malignancy affecting the liver and is the fourth most common cause of cancer‐related deaths worldwide.^1^, ^2^ It largely arises from a combination of sustained insults to the liver including chronic inflammatory changes and necrosis of hepatocytes, fibrosis and cirrhosis of the liver, over a median lag time of ~10–50 years, depending on the underlying aetiology.^3^, ^4^ Traditionally, the highest prevalence of HCC worldwide mainly affects lower‐to‐middle income countries (LMICs) in areas such as East and South‐East Asia and Africa, although the incidence of HCC is also rising in developed areas including the United States, Europe and Japan.^2^, ^3^

Common underlying aetiological factors for HCC include chronic hepatitis B and/or C virus (HBV/HCV) infection, which still represent the most prevalent causes of HCC, especially in LMICs.^2^ However, other more lifestyle‐associated causes such as alcohol‐related liver disease, non‐alcoholic fatty liver disease/non‐alcoholic steatohepatitis (NAFLD/NASH), metabolic syndrome and diet‐related factors (e.g. aflatoxins), along with genetic factors and predispositions (e.g. hereditary haemochromatosis, α1‐anti‐trypsin deficiency, etc.) also play a significant role in the pathogenesis of HCC.^2^, ^5^, ^6^ Such a variety of underlying aetiologies pose both an opportunity and a challenge in preventing HCC from a public health perspective and in treating and managing the overall rising numbers of cases worldwide.^2^, ^6^ For example, the incidence of cases of HCC is rising in the United States mostly as a result of increasing levels of alcoholic cirrhosis and NAFLD/NASH, whereas the incidence of cases in large parts of Asia and Africa where chronic HBV and HCV infections remain the most common cause are steady or falling due to increased uptake of immunisations and use of antiviral therapies.^5^, ^7^, ^8^

**FIGURE 1:**
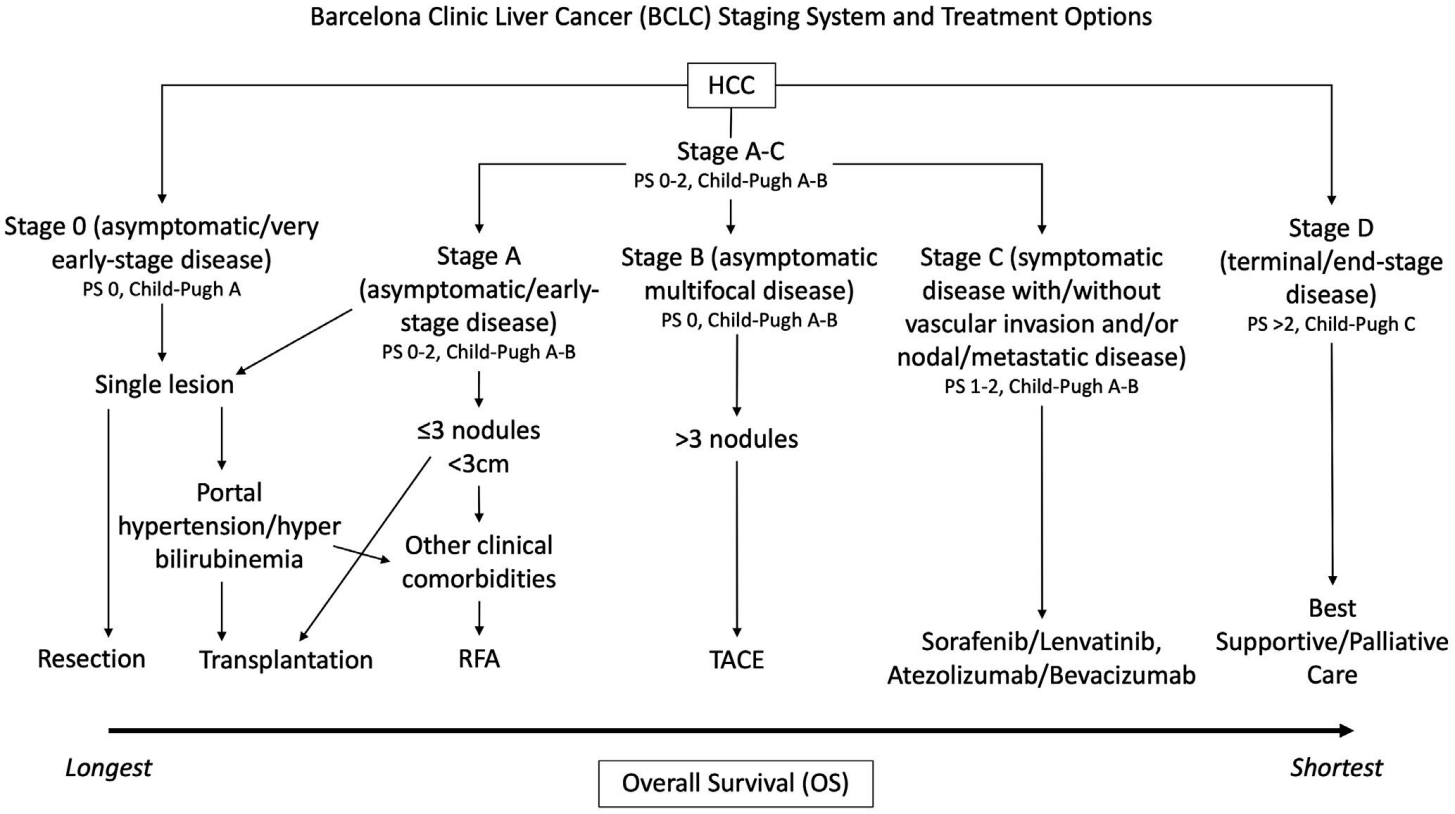
Treatment options for hepatocellular carcinoma (HCC) according to the Barcelona Clinic Liver Cancer (BCLC) staging system. Some centres use carefully regulated extended criteria regarding size and number of tumours to select for transplantation. More invasive treatments for early stage disease generally have a better prognosis compared to less invasive/palliative treatments for advance disease. Adapted from Tellapuri et al.^13^ PS, performance status; RFA, radiofrequency ablation; TACE, transarterial chemoembolisation

Treatment options for HCC are dependent on the stage of the disease (Figure 1). These include surgery (including curative resection and liver transplantation), radiofrequency ablation, stereotactic ablative radiotherapy/stereotactic body radiation therapy, transarterial chemoembolisation (TACE), transarterial radioembolisation, and systemic targeted therapies including Atezolizumab/Bevacizumab (immune checkpoint inhibitors [ICIs] that are now the established standard of care for advanced HCC) and Sorafenib/Lenvatinib (multi‐targeted tyrosine kinase inhibitors that were previously the standard of care for advanced HCC).^9^, ^10^, ^11^ However, the curative treatment options for HCC (i.e. surgical) are often only reserved for a minority of individuals presenting with localised HCC, given delays in presentation and poor performance status/liver function reserve.^9^ This is partly due to the fact that individuals with HCC are sometimes asymptomatic for many months or years, often diagnosed as an incidental radiological finding, which is compounded by a lack of evidence for an established generalised cost‐effective screening programme worldwide.^1^, ^12^

For patients with advanced HCC, licensed systemic multi‐targeted kinase inhibitors, including Sorafenib and Lenvatinib, are sometimes poorly tolerated due to their well‐characterised side‐effect profiles and provide minimal benefits in overall survival.^14^, ^15^, ^16^, ^17^ Moreover, the rates of recurrence of HCC have historically been shown to be ~10%–20% following liver transplantation and are associated with a poorer prognosis.^18^, ^19^, ^20^, ^21^ All of this in combination leads to a limited 5‐year survival rate (~18%) for HCC and the need for new and alternative treatments to be developed.^1^, ^5^, ^22^

**FIGURE 2:**
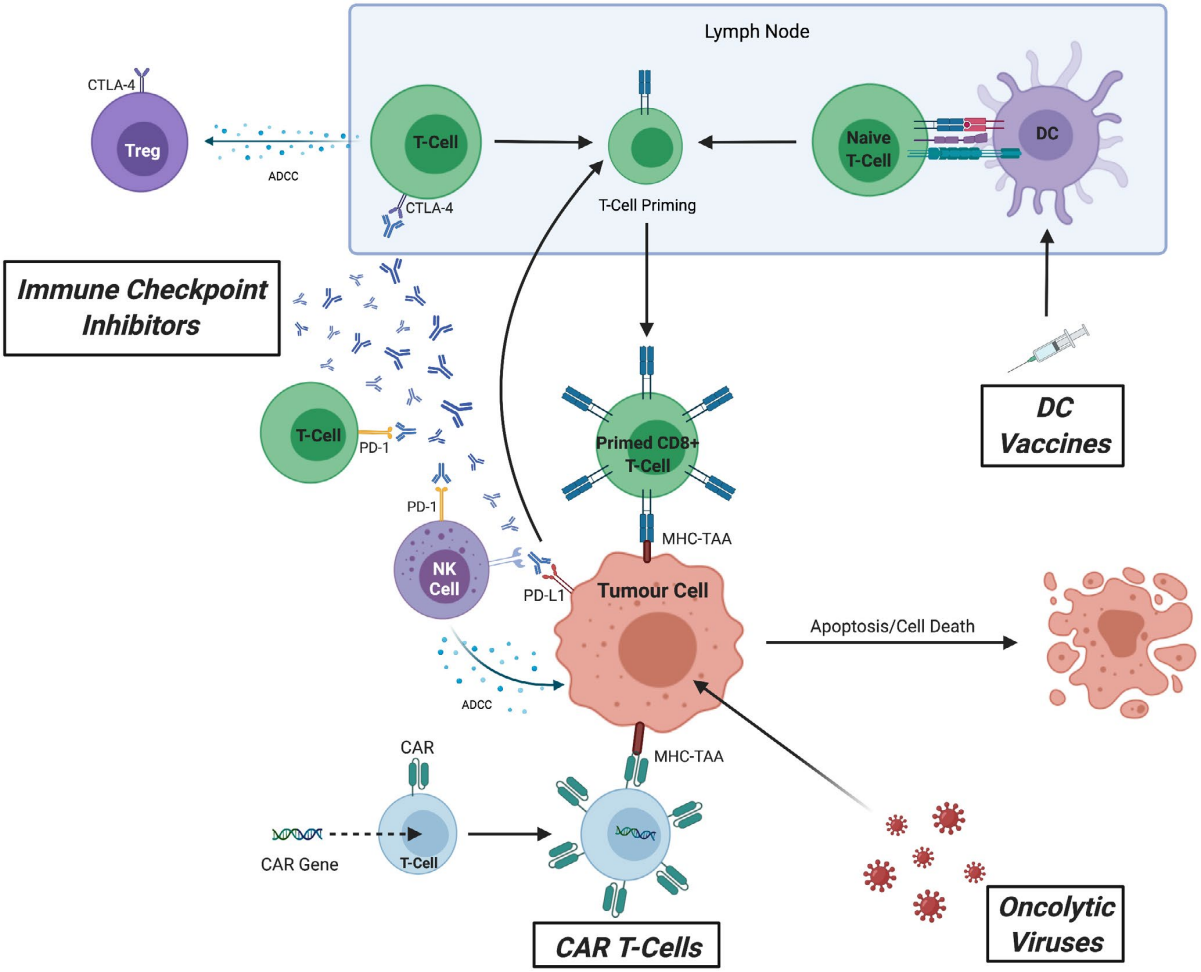
The multifaceted actions of cancer immunotherapy including immune checkpoint inhibitors (ICIs), chimeric antigen receptor (CAR) T‐cells, dendritic cell (DC) vaccines and oncolytic viruses (OVs). Each mode of cancer immunotherapy aims to modulate an individual's immune response against tumour cells, either directly or indirectly, through priming and stimulation to enhance the autologous effect of antitumour activity towards the tumour and inducing tumour apoptosis/cell death or by modifying the surrounding tumour microenvironment to promote immunogenicity. ICIs consist of monoclonal antibodies which target negative immune checkpoint costimulatory molecules expressed on both innate and adaptive immune cells (CTLA‐4, programmed death cell protein 1 [PD‐1], programmed death‐ligand 1 [PD‐L1]) and tumour cells (PD‐L1). These serve to directly inhibit the negative interaction between tumour cells and surrounding host immune cells (through blocking PD‐1/PD‐L1 interaction and CTLA‐4) which upregulates T‐cell priming in the lymph nodes and increases recognition of tumour cells by primed CD8^+^ T‐cells through major histocompatibility complex (MHC) recognition of tumour‐associated antigens (TAAs) expressed on the surface of tumour cells. Moreover, ICIs also promote antibody‐dependent cellular cytotoxicity (ADCC) against T regulatory (Treg) cells (which primarily serve to downregulate the immune response against tumour cells) and tumour cells themselves through NK cell‐dependent ADCC. CAR T‐cells are exogenously engineered T‐cells expressing a specialised CAR that target specific TAAs to promote a controlled positive downstream immune response. DC vaccines consist of isolated autologous DCs that are primed in vitro against TAAs before being reintroduced into individuals to promote host T‐cell priming. OVs comprise of genetically engineered inactivated viruses that preferentially infect tumour cells and upregulate both humoral and cell‐mediated immune responses^25^, ^26^, ^27^, ^28^

Cancer immunotherapy has revolutionised the treatment of solid malignancies. Immunotherapy aims to harness an individual's immune system to selectively target and kill tumour cells.^23^, ^24^ This can be achieved via a variety of mechanisms, as illustrated in Figure 2. This review summarises various immunotherapies both currently licensed for use in the treatment of HCC and those in the stages of preclinical and clinical development.

## 2 IMMUNE CHECKPOINT INHIBITORS

**FIGURE 3:**
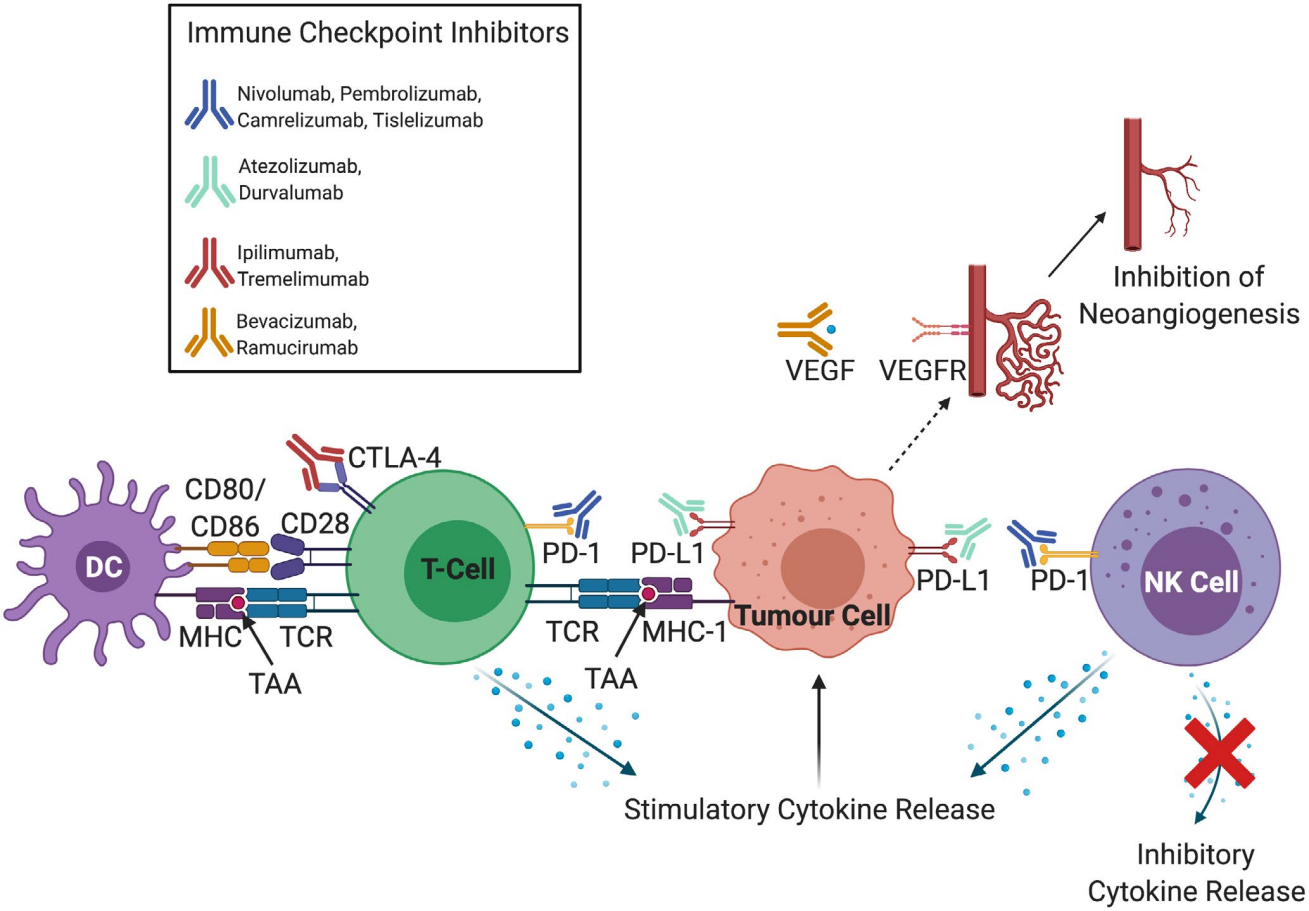
The multitude of effects of immune checkpoint inhibitors that have been developed targeting molecular drivers of hepatocellular carcinoma progression. Targets include receptor/ligand pairs found on innate immune cells including natural killer (NK) cells (e.g. programmed death cell protein 1 [PD‐1]), adaptive immune cells including T‐cells (e.g. PD‐1, cytotoxic T‐lymphocyte‐associated protein 4 [CTLA‐4]), and those found on tumour cells themselves (e.g. PD‐L1).

The use of ICIs in the treatment of various cancers has stimulated marked improvements in the treatment of solid malignancies.^29^, ^30^ ICIs broadly target negative immune costimulatory modulators expressed on the cellular surfaces of both antigen‐presenting cells (APCs) and T‐cells (Table 1). These receptor‐ligand pairs downregulate the effects of T‐cell priming and activation, in order to keep the adaptive immune system ‘in‐check’, with a more minor effect on other immune cell types.^31^, ^32^ Tumour cells manipulate immune checkpoints by upregulating negative costimulatory molecules, through their own intrinsic tumour activity, or by altering aspects of the surrounding tumour microenvironment to enhance immune evasion, tumour progression and spread.^33^Therefore, ICIs aim to block the effects of negative immune costimulatory molecules, in order to unleash antitumour activity through promotion and upregulation of T‐cell priming and activation.^31^, ^32^ ICIs tip the balance away from a relative immunosuppressive tumour microenvironment and inhibitory cytokines, towards promotion of an immune‐mediated response against tumour cells and stimulatory cytokine release, in order to reduce the rate of progression and spread of tumour cells (Figure 3).^29^, ^30^, ^31^, ^32^, ^33^ There have been several licensed ICIs approved for the treatment of cancers including metastatic melanoma, non‐small cell lung cancer, renal cell carcinoma, and colorectal cancer (with microsatellite instability).^30^ They primarily target key negative immune costimulatory modulators expressed on either APCs or T‐cells. These include programmed death cell protein 1 (PD‐1) (primarily expressed on T‐cells), programmed death‐ligand 1 (PD‐L1) (expressed on APCs, other immune cells and tumour cells), and cytotoxic T‐lymphocyte‐associated protein 4 (CTLA‐4) (primarily expressed on T‐cells).^34^, ^35^

**TABLE 1:** Summary of the main clinical trials, both completed and ongoing, involving ICIs both as monotherapies and in combination with other locoregional/systemic therapies used to treat hepatocellular carcinoma (HCC), the targeted disease stage, reported primary outcomes, and the most common and/or serious (grade ≥ 3) treatment‐related adverse events (TRAEs), compared to Sorafenib/Lenvatinib (where possible)

Clinical trials in HCC involving ICIs	Phase	Disease stage targeted	Comparison arms	Patient numbers	Outcomes	Adverse events	Publication
*ICI monotherapy*							
CheckMate 040 (NCT01658878)	I/II	Advanced HCC, Child‐Pugh class A, previously treated with or naïve/intolerant to Sorafenib	Nivolumab	262	Cohort 1 (dose escalation) = ORR 15%, mDRR17 months, 9‐month OS rate 66%, mOS 15 months Cohort 2 (dose expansion) = ORR 20%, mDRR 9.9 months, 9‐month OS rate 74%, 9‐month PFS 28%	Cohort 1 (dose escalation) – grade ¾ TRAE rate 25%, therapy discontinuation rate 6%, most common grade TRAEs: rash (23%), AST increase (21%), lipase increase (21%) Cohort 2 (dose expansion) – grade 3/4 TRAE rate 19%, therapy discontinuation rate 11%, most common TRAEs: fatigue (23%), pruritis (21%), rash (15%)	El‐Khoueiry et al.^50^
CheckMate 459 (NCT02576509)	III	Advanced HCC, Child‐Pugh class A, Sorafenib‐naïve	Nivolumab vs. Sorafenib	743	mOS 16.4 months (HR 0.85, *p* = 0.0752), ORR 15%, mPFS 3.7 months, 24‐month OS rate 36.8%	Grade 3/4 TRAE rate: Nivolumab 34% vs. Sorafenib 49% Therapy discontinuation rate: Nivolumab 4% vs. Sorafenib 8%	Yau et al.^51^
KEYNOTE‐224 (NCT02702414)	II	Advanced HCC, BCLC stage B or C, Child Pugh class A, previous Sorafenib failure/intolerance	Pembrolizumab	104	ORR 17%, mOS 12.9 months, mPFS 4.9 months, 12‐month OS rate 54%, 12‐month PFS rate 28%	Grade ≥ 3 TRAE rate 26% Therapy discontinuation rate following an adverse event 5% Most common TRAEs: fatigue (21%), AST increase (13%), pruritis (12%), diarrhoea (11%)	Zhu et al.^54^
KEYNOTE‐240 (NCT02702401)	III	Advanced HCC, BCLC stage B or C, Child‐Pugh class A, previous Sorafenib failure/intolerance	Pembrolizumab vs. placebo	413	OS (HR 0.781, one‐sided *p* = 0.0238*), PFS (HR 0.78, one‐sided *p* = 0.0209*), mOS 13.9 months, mPFS 3 months, ORR 18.3%	Grade ≥ 3 TRAE rate: Pembrolizumab 18.6% vs. placebo 7.5%	Finn et al.^56^, ^57^
KEYNOTE‐394 (NCT03062358)	III	Advanced HCC, BCLC stage B or C, Child‐Pugh class A, previous Sorafenib failure/intolerance, Asian ethnicity	Pembrolizumab vs. placebo	454	Ongoing		
NCT02989922	II	Advanced HCC, BCLC stage B or C, Child‐Pugh class A, previous systemic therapy failure/intolerance, high HBV prevalence	Camrelizumab	220	ORR 14.7%, 6‐month OS rate 74.4%, mOS 13.8 months, mPFS 2.1 months	Grade 3/4 TRAE rate 22% Therapy discontinuation rate following a TRAE 4% Most common TRAEs: RCCEP (67%), AST increase (25%), ALT increase (24%), proteinuria (23%)	Qin et al.^60^
RATIONALE 301 (NCT03412773)	III	Unresectable HCC, BCLC stage B or C, Child‐Pugh class A, Sorafenib‐naïve	Tislelizumab vs. Sorafenib	674	Ongoing		Qin et al.^61^
REACH (NCT01140347)	III	Unresectable HCC, BCLC stage B or C, Child Pugh class A, previous Sorafenib failure/intolerance	Ramucirumab vs. placebo	565	mOS 9.2 months (HR 0.87, *p* = 0.14), mPFS 2.8 months (HR 0.63, *p* < 0.0001), 12‐month OS rate 39.7%, 12‐month PFS rate 20.7%, ORR 7% (*p* < 0.0001)	Grade ≥ 3 TRAE rate: Ramucirumab 36% vs. placebo 29% Therapy discontinuation rate following an adverse event 10% Most common TRAEs: peripheral oedema (36%), ascites (22%), headache (18%)	Zhu et al.^62^
REACH‐2 (NCT02435433)	III	Unresectable HCC, BCLC stage B or C, Child Pugh class A, previous Sorafenib failure/intolerance, α‐fetoprotein > 400 ng/ml	Ramucirumab vs. placebo	292	mOS 8.5 months (HR 0.71, *p* = 0.0199), mPFS 2.8 months (HR 0.452, *p* < 0.0001), ORR 5% (*p* = 0.1697)	TRAE rate: Ramucirumab 11% vs. placebo 5% Therapy discontinuation rate following a TRAE 11% Most common TRAEs: fatigue (27%), peripheral oedema (25%), hypertension (25%)	Zhu et al.^63^
*Combination ICI therapy*							
IMbrave150 (NCT03434379)	III	Advanced/unresectable HCC, Child‐Pugh class A, Sorafenib‐naïve	Atezolizumab+Bevacizumab vs. Sorafenib	501	OS (HR 0.58, *p* < 0.001), mOS 19.2 months (HR 0.66, *p* = 0.0009), mPFS 6.8 months (HR 0.59, *p* < 0.001), ORR 27.3% (*p* < 0.001)	Grade 3/4 TRAE rate: Atezolizumab+Bevacizumab 56.5% vs. Sorafenib 55.1% Grade 5 TRAE rate: Atezolizumab+Bevacizumab 4.6% vs. Sorafenib 5.8% Therapy discontinuation rate: Atezolizumab+Bevacizumab 15.5% vs. Sorafenib 10.3% Most common grade 3/4 TRAEs: hypertension (15.2%), AST increase (7%), ALT increase (3.6%)	Finn et al.^80^, ^82^
RESCUE (NCT03463876)	II	Advanced HCC, BCLC stage B or C, Child‐Pugh class A, previous Sorafenib failure/intolerance	Camrelizumab+Apatinib	190	Ongoing		
NCT04035876	I/II	Early‐stage HCC amenable to liver transplantation	Camrelizumab+Apatinib	120 (est)	Ongoing		
CheckMate 040 (NCT01658878)	I/II	Advanced HCC, Child‐Pugh class A, previous Sorafenib failure/intolerance	Nivolumab+Ipilimumab (3 dosing arms)	148	Arm 1 = ORR 32%, mDRR 17.5 months Arm 2 = ORR 31%, mDRR 22.2 months Arm 3 = ORR 31%, mDRR 16.6 months	Grade 3/4 TRAE rate: arm 1 53%, arm 2 29%, arm 3 31%; therapy discontinuation rate: arm 1 18%, arm 2 6%, arm 3 2%; most common TRAEs (across all arms): rash, pruritis, diarrhoea, hepatitis, AST increase	Yau et al.^84^
CheckMate 9DW (NCT04039607)	III	Advanced HCC, Child‐Pugh class A	Nivolumab+Ipilimumab vs. Sorafenib/Lenvatinib	650 (est)	Ongoing		
HIMALAYA (NCT03298451)	III	Unresectable HCC, BCLC stage B or C, Child‐Pugh class A	Durvalumab+Tremelimumab vs. Durvalumab vs. Sorafenib	1504 (est)	Ongoing		Abou‐Alfa et al.^85^
LEAP‐002 (NCT03713593)	III	Unresectable HCC, BCLC stage B or C, Child‐Pugh class A	Pembrolizumab+Lenvatinib vs. Lenvatinib	750 (est)	Ongoing		Llovet et al.^91^
NCT03418922	Ib	Unresectable HCC, BCLC stage B or C, Child‐Pugh class A	Nivolumab+Lenvatinib	30	ORR 76.7%	Therapy discontinuation rate following a TRAE: Nivolumab 13.3%, Lenvatinib 6.7% Most common TRAEs: Palmar‐plantar erythrodysesthesia (56.7%), dysphonia (53.3%)	Kudo et al.^94^
NCT03841201	II	Advanced HCC, Child‐Pugh class A	Nivolumab+Lenvatinib	50 (est)	Ongoing		
COSMIC‐312 (NCT03755791)	III	Advanced HCC, BCLC stage B or C, Child‐Pugh class A	Atezolizumab+Cabozantinib vs. Sorafenib	740 (est)	Ongoing		Kelley et al.^92^
*ICIs in combination with other systemic/locoregional therapies*							
IMMULAB (NCT03753659)	II	Intermediate/multifocal HCC, Child‐Pugh class A	Pembrolizumab+RFA/MWA/brachytherapy+TACE vs. Pembrolizumab+RFA/MWA/brachytherapy	30 (est)	Ongoing		Vogel et al.^96^
IMMUTACE (NCT03572582)	II	Intermediate/multifocal HCC, Child‐Pugh class A	Nivolumab+TACE	49	Ongoing		
PETAL (NCT03397654)	I/II	Intermediate HCC, BCLC stage B, Child‐Pugh class A	Pembrolizumab +TACE	26 (est)	Ongoing		Pinato et al.^98^
NCT02821754	II	Advanced HCC, BCLC stage B or C, Child‐Pugh class A/B7, previous Sorafenib failure/intolerance	Durvalumab+Tremelimumab+RFA/TACE/cryoablation vs. Durvalumab+Tremelimumab	10	mPFS 7.8 months, mOS 15.9 months		Floudas et al.^97^
NCT03638141	II	Intermediate HCC	Durvalumab +Tremelimumab + TACE	30 (est)	Ongoing		
NCT03937830	II	Advanced HCC, BCLC stage B or C, Child‐Pugh class A	Durvalumab+Tremelimumab+Bevacizumab+TACE	22 (est)	Ongoing		
EMERALD‐1 (NCT03778957)	III	Intermediate HCC, Child‐Pugh class A/B7	Durvalumab+Bevacizumab+TACE vs. Durvalumab+TACE vs. TACE	710 (est)	Ongoing		Sangro et al.^99^
TRIPLET (NCT04191889)	II	Advanced HCC, BCLC stage C, Child‐Pugh class A/B7, no previous systemic therapy	Camrelizumab+Apatinib+HAIC	84 (est)	Ongoing		
NCT03092895	II	Advanced HCC, Child‐Pugh class A/B7	Camrelizumab+FOLFOX4/GEMOX	34	ORR 26.5%	Grade ≥ 3 TRAE rate 85.3% Most common grade ≥ 3 TRAEs: neutropenia (55.9%), leukopenia (38.2%), thrombocytopenia (17.6%)	Qin et al.^100^
NCT03316872	II	Advanced HCC, Child‐Pugh class A, previous Sorafenib failure/intolerance	Pembrolizumab+SBRT	30 (est)	Ongoing		
NCT03482102	II	Advanced/unresectable HCC, Child‐Pugh class A, previous Sorafenib failure/intolerance	Durvalumab+Tremelimumab+SBRT	70 (est)	Ongoing		
CheckMate 9DX (NCT03383458)	III	Resected HCC/complete response following ablation, Child‐Pugh class A, high risk of HCC recurrence	Nivolumab+curative resection/RFA vs. curative resection/RFA	530 (est)	Ongoing		
EMERALD‐2 (NCT03847428)	III	Resected HCC/complete response following ablation, Child‐Pugh class A, high risk of HCC recurrence	Durvalumab+Bevacizumab+curative resection/RFA vs. Durvalumab+curative resection/RFA vs. curative resection/RFA	888 (est)	Ongoing		Knox et al.^101^

Abbreviations: ALT, alanine aminotransferase; AST, aspartate aminotransferase; BCLC, Barcelona Clinic Liver Cancer; DRR, durable response rate; HAIC, hepatic arterial infusion chemotherapy; HBV, hepatitis B virus; HR, hazard ratio; ORR, objective response rate; OS, overall survival; RFA, radiofrequency ablation; MWA, microwave ablation; PFS, progression‐free survival; RCCEP, reactive cutaneous capillary endothelial proliferation; SBRT, stereotactic body radiation therapy; TACE, transarterial chemoembolisation; m, median.

* Non‐statistically significant.

## 3 MODULATION OF MONOCLONAL ANTIBODY EFFECTOR FUNCTIONS THROUGH FC ENGINEERING

Targeted therapies that selectively bind to and inhibit the activity of these co‐inhibitory receptors/ligands predominantly exist in the form of recombinant monoclonal antibodies. For example, Nivolumab (a fully humanised IgG4 monoclonal antibody) and Pembrolizumab (a humanised IgG4 monoclonal antibody) target and bind to PD‐1; Atezolizumab (a fully humanised IgG1 monoclonal antibody) targets and binds to PD‐L1; and Ipilimumab (a fully humanised IgG1 monoclonal antibody) targets and binds to CTLA‐4.^9^, ^30^, ^32^ The reason why IgG isotypes are selected—specifically IgG1, IgG2 and IgG4—are for their long half‐life, stability and ability to more accurately predict, and thus provide better control of, downstream effector functions to allow for optimal potentiation of monoclonal antibody action.^36^, ^37^, ^38^ For monoclonal antibodies targeting PD‐1 and PD‐L1, they are also specifically engineered with single or multiple point mutations in their Fc regions to reduce the interaction between the Fc portion of the monoclonal antibody and effector cells expressing Fcγ receptors. This reduces antibody‐dependant cellular cytotoxicity (ADCC), antibody‐dependant cellular phagocytosis and complement‐dependant cytotoxicity against host immune effector cells.^39^, ^40^, ^41^ For monoclonal antibodies targeting CTLA‐4, their primary function is to increase ADCC against T regulatory cells (Tregs) (Table 2).^39^, ^41^

**TABLE 2:** A list of the monoclonal antibodies that have been developed or are currently in development that serve as ICIs targeting PD‐1, PD‐L1 and CTLA‐4 in the treatment of HCC. The majority of them are specifically selected for their antibody class and genetic engineering through single or multiple point mutations in their Fc region in order to reduce unwanted off‐target or cytotoxic side effects. This is primarily due to a reduction (↓) or complete abrogation (↓↓↓) in the interaction between the Fc portion of the monoclonal antibody and effector cells expressing Fcγ receptors (FcγRs) which, in turn, reduces antibody‐dependant cellular cytotoxicity (ADCC), antibody‐dependant cellular phagocytosis (ADCP) and complement‐dependant cytotoxicity (CDC). Ipilimumab and Tremelimumab both target CTLA‐4 which upregulates (↑) ADCC against Treg cells, with no discernible effects on Fc/FcγR interaction (given that they are both wild‐type monoclonal antibodies) or ADCP/CDC in human models(‐).

Monoclonal antibody	Ligand target	Antibody class	Fc engineering	Fc/FcγR interaction	Mechanism of action		
					ADCC	ADCP	CDC
Nivolumab	PD‐1	IgG4	S228P	↓	↓	↓	↓
Pembrolizumab	PD‐1	IgG4	S228P	↓	↓	↓	↓
Camrelizumab	PD‐1	IgG4	S228P	↓	↓	↓	↓
Tislelizumab	PD‐1	IgG4	S228P/E233P/F234V/L235A/D265A/R409K	↓↓↓	↓	↓	↓
Atezolizumab	PD‐L1	IgG1	N298A	↓↓↓	↓	↓	↓
Durvalumab	PD‐L1	IgG1	L234F/L235E/P331S	↓↓↓	↓	↓	↓
Ipilimumab	CTLA‐4	IgG1	Wild‐type	‐	↑	‐	‐
Tremelimumab	CTLA‐4	IgG2	Wild‐type	‐	↑	‐	‐

## 4 TREATMENT OF HCC WITH ICIs

The liver naturally exists in a relatively immunosuppressed state which is heavily regulated by the immune system given its physiological role in processing foreign materials and first‐pass metabolism. This state of immunosuppression is hijacked and further exacerbated by HCC in order to promote immune evasion and tumour cell survival. Therefore, there exists a potential opportunity for ICIs to operate within this niche and reverse this process in the tumour, whilst maintaining immunosuppression in the background liver in order to avoid hepatic autoimmunity. Several clinical trials have previously been undertaken or are currently in progress exploring the impact of ICIs in treating HCC at different stages and with different underlying aetiologies, either as a monotherapy or in various combinations (Table 1).^42^, ^43^, ^44^, ^45^, ^46^, ^47^, ^48^, ^49^

### 4.1 Nivolumab

There have been several clinical trials investigating the use of ICIs as monotherapies in the first or second‐line setting (following failure of Sorafenib/Lenvatinib) for advanced HCC.^44^, ^46^, ^48^, ^49^ These have so far primarily been with monoclonal antibodies that target PD‐1.^44^, ^46^, ^48^ The CheckMate 040 (NCT01658878) and CheckMate 459 (NCT02576509) trials studied the use of Nivolumab for the treatment of histologically confirmed advanced HCC with/without HBV/HCV infections.^50^, ^51^ The CheckMate 040 trial looked at the safety and clinical efficacy of different doses of Nivolumab in 262 patients (who had previously received or not received Sorafenib) in multiple centres/regions worldwide. The results found overall objective response rates (ORR) of 15% and 20% in the dose‐escalation phase and dose‐expansion phase respectively, with an overall median overall survival (mOS) of 15 months in the dose‐expansion phase. The reduction in tumour burden was greatest in those individuals whose disease was not driven by viral hepatitis and those who had not previously received Sorafenib.^50^ Moreover, the study also demonstrated an acceptable safety and drug tolerability profile which led to Nivolumab being granted regulatory approval by the United States Food and Drug Administration (FDA) in 2017 for the treatment of advanced HCC following failure of first‐line Sorafenib therapy.^44^, ^46^, ^47^, ^49^, ^50^ This was followed by the CheckMate 459 trial which was a multicentre phase III trial that directly compared the effects of Nivolumab against Sorafenib in advanced HCC. Although the study did not achieve a statistically significant difference in mOS (HR 0.85, *p* = 0.0752) (its primary endpoint), clinical benefits of Nivolumab, including mOS and ORR, were observed in all subgroups of patients including locally advanced and metastatic disease, underlying aetiologies (viral hepatitis vs. non‐viral hepatitis) and regions (Asia vs. non‐Asia) compared to Sorafenib, together with an adequate adverse event profile.^51^ However, following results from the CheckMate 459 trial, Nivolumab monotherapy for the second‐line treatment of advanced HCC was recently withdrawn for use by the FDA due to its failure to achieve a statistically significant primary endpoint.^52^

### 4.2 Pembrolizumab

Another PD‐1 inhibitor that has been widely studied for the treatment of advanced HCC is Pembrolizumab.^42^, ^43^, ^44^, ^45^, ^46^, ^47^, ^48^, ^49^ The KEYNOTE‐224 (NCT02702414) trial was a multicentre nonrandomised phase II trial assessing the clinical safety and efficacy of Pembrolizumab 104 patients with advanced progressive HCC of any underlying aetiology and previously treated with Sorafenib (either up to failure of treatment or stopped due to drug intolerances/adverse effects). The study found an ORR of 17% for an estimated minimum duration of 9 months, with a manageable adverse event profile.^53^, ^54^ This subsequently led to regulatory approval of Pembrolizumab for second‐line treatment of advanced HCC by the FDA.^42^, ^43^, ^44^, ^45^, ^46^, ^47^, ^49^ This was followed by the KEYNOTE‐240 trial (NCT02702401), a randomised phase III trial comparing Pembrolizumab to placebo plus best‐supportive care following treatment with Sorafenib.^55^, ^56^, ^57^ Similarly to Nivolumab, this study did not meet its predefined primary outcome in terms of achieving statistical significance (HR 0.781, one‐sided *p* = 0.0238), but did show clinical benefits in mOS, progression‐free survival (PFS) and ORR, particularly in the Asian subgroup of patients.^55^, ^56^, ^57^, ^58^ This is furthered by the recent announcement of the preliminary results of the KEYNOTE‐394 trial (NCT03062358), which assessed the clinical efficacy of Pembrolizumab in Asian patients with advanced HCC previously treated with Sorafenib versus placebo, indicating statistically significant improvements in OS (the primary endpoint), PFS and ORR,^59^ thus again showing potential benefits in this subgroup of patients.

### 4.3 Camrelizumab

One of the most recent PD‐1 monoclonal antibodies that have been approved for regulatory use in China for the treatment of advanced HCC in patients who previously failed first‐line treatment is Camrelizumab.^49^ This was based on the results of a randomised phase II trial (NCT02989922) which demonstrated an ORR of 14.7% and an mOS of 13.8 months in patients with predominantly HBV‐driven advanced HCC, with follow‐up currently ongoing.^60^

## 5 PATIENT SELECTION FOR ICI MONOTHERAPIES

One of the possibilities as to why both the CheckMate 459 and the KEYNOTE‐240 trials did not reach their clinically predefined primary outcome endpoints was due to the fact that both trials were unable to account for the regulatory approval of the use of other second‐line targeted therapies for the treatment of advanced HCC, including Regorafenib (a multi‐target tyrosine kinase inhibitor) which was licensed for use by the FDA in 2017, whilst both clinical trials were still ongoing.^42^, ^46^, ^49^, ^51^, ^56^ However, certain patient subgroups may have benefited from anti‐PD‐1 therapy more than others.^51^, ^56^ Significant clinical improvements in OS were observed in patients receiving Nivolumab who were of Asian ethnicity, positive for HBV/HCV and had evidence of metastatic spread,^51^ and in patients receiving Pembrolizumab who was also of Asian ethnicity (excluding Japan), were HBV‐positive and had disease progression despite Sorafenib.^56^ Therefore, further work needs to be undertaken to better identify molecular biomarkers predictive for patient benefit. This approach was demonstrated in the REACH (NCT01140347) and REACH‐2 (NCT02435433) trials, where Ramucirumab (a monoclonal antibody targeting the VEGF2 receptor) was shown to have increased overall survival benefits in patients with advanced HCC who had elevated serum concentrations of α‐fetoprotein (AFP) (>400 ng/ml),^62^, ^63^ which ultimately led to its regulatory approval by the FDA for second‐line treatment of advanced HCC.^42^, ^43^, ^45^, ^46^, ^47^, ^49^

One of the more recent ways in which patients are potentially stratified based on predictive response to immunotherapy is through measuring expression of specific molecular biomarkers on tumour cells.^34^, ^35^ There are currently no clinically established molecular biomarkers for advanced HCC that can predict an outcome in relation to treatment with ICIs.^42^, ^43^, ^46^, ^47^, ^48^, ^49^ One of the most widely studied predictive molecular biomarkers in PD‐1 and PD‐L1 immunotherapy is the level of expression of PD‐L1, which has previously been shown to correlate with survival outcomes in a variety of other cancers, including melanoma, non‐small cell lung cancer and head and neck squamous cell carcinoma.^34^, ^35^ However, results from both the CheckMate 040 and KEYNOTE‐224 trials demonstrated non‐statistically significant associations between the levels of expression of PD‐L1 and survival outcomes.^50^, ^53^ Further research into characterising a potential role for PD‐L1 expression and establishing other molecular biomarkers in predicting a treatment response for ICIs in HCC is warranted.^42^, ^43^, ^46^, ^47^, ^48^, ^49^

## 6 MECHANISMS OF ICI RESISTANCE IN HCC

Immune checkpoint inhibitor monotherapy in the treatment of advanced HCC may be limited by multiple treatment‐resistance mechanisms.^64^, ^65^, ^66^, ^67^ These mechanisms can broadly be divided into tumour intrinsic factors and tumour extrinsic factors. Tumour intrinsic factors include upregulation of multiple oncogenic cell signalling pathways (through activating mutations), epigenetic modifications, and expression of cytokines that promote tumour cell proliferation and immune evasion.^64^, ^65^, ^66^, ^67^ Oncogenic cell signalling pathways that are upregulated in HCC include receptor tyrosine kinases (EGFR, FGFR, c‐MET, VEGFR, etc.), Wnt/ß‐catenin, MAPK/ERK, PI3K/AKT/mTOR, and the JAK/STAT signalling pathways.^68^, ^69^, ^70^, ^71^, ^72^ They are associated with decreased intratumoural infiltration and effector function of T‐cells and dendritic cells (DCs), and increased T‐cell exhaustion.^64^, ^65^, ^66^, ^67^ Furthermore, cytokines such as interleukin (IL)‐10 and TGF‐ß promote tumour cell proliferation and immune evasion through a reduction in the recruitment of APCs and antigen presentation, along with overexpression of alternative co‐inhibitory cell‐surface ligands such as T‐cell immunoglobulin domain and mucin domain 3 and lymphocyte‐activation gene 3.^64^, ^65^, ^66^, ^67^ The HCC tumour microenvironment is able to further promote immune evasion/escape and subsequent tumour cell survival and spread,^47^, ^48^, ^73^ via mechanisms that are both generic to many tumour types and additionally HCC‐specific mechanisms.^74^, ^75^, ^76^, ^77^ HCC is a tumour that is characterised by fibrotic changes and chronic inflammation (as a result of liver cirrhosis) which creates a physical barrier for immune cell infiltration as well as secretion of proinflammatory cytokines that downregulate immune cell function.^74^, ^75^, ^76^, ^77^ This is contained in a surrounding microenvironment that is largely hypoxic, giving the liver a unique metabolic profile, and immune tolerogenicity (given the large amounts of foreign materials that pass through the liver during first‐pass metabolism), thus further exacerbating a largely immunosuppressive environment.^74^, ^75^, ^76^, ^77^ Cytokines such as IL‐10, TGF‐ß, VEGF and hepatocyte growth factor are produced by hepatic stromal cells including Kupffer cells and hepatic stellate cells to reduce the recruitment and activity of tumour‐infiltrating lymphocytes (TILs) and upregulate the production and activity of tumour‐associated macrophages, Tregs and myeloid‐derived suppressor cells (MDSCs).^48^, ^73^, ^74^, ^75^, ^76^ ICI activity and efficacy rely on the ability of the host to generate a desired immune response against the tumour cells.^64^, ^65^, ^66^, ^67^ Therefore, those tumour cells which express alternative TAAs or have a low tumour mutational burden may not respond to ICIs from the outset—termed innate/primary resistance.^64^, ^65^, ^66^, ^67^ Alternatively, an individual may develop resistance to ICIs over time due to the effects of the immunosuppressive tumour microenvironment gradually overwhelming the activity of ICIs rendering their use redundant—termed acquired/secondary resistance.^64^, ^65^, ^66^, ^67^ More work is required to elucidate further key cell signalling pathways and better understand the role of the development of tumour resistance in advanced HCC, particularly in the context of ICIs.^43^, ^47^ This also provides justification for several clinical trials that have been or are currently being undertaken combining different treatments for HCC including targeted therapies with existing locoregional therapies and immunotherapy for advanced HCC, which is postulated to reduce the emergence of tumour resistance^44^, ^47^, ^48^ and to improve synergistic benefits.^78^, ^79^

## 7 PD‐1/PD‐L1 INHIBITION WITH ANTI‐VEGF THERAPY

To date, one of the most successful immunotherapy combinations developed to treat advanced HCC has been Atezolizumab (a PD‐L1 inhibitor) and Bevacizumab (a circulating VEGF inhibitor). The IMbrave150 trial (NCT03434379) was a global multicentre randomised phase III clinical trial that recruited 501 patients and compared OS and PFS (the co‐primary endpoints) of Atezolizumab and Bevacizumab against Sorafenib.^80^, ^81^ There was a 42% reduction in the risk of death with Atezolizumab and Bevacizumab in comparison to Sorafenib (HR 0.58, *p* < 0.001).^80^, ^81^ mOS was reported to be 19.2 months in patients receiving Atezolizumab and Bevacizumab compared to 13.4 months in patients receiving Sorafenib (HR 0.66, *p* = 0.0009).^82^ mPFS was 6.8 months (Atezolizumab and Bevacizumab) versus 4.3 months (Sorafenib) (HR 0.59, *p* < 0.001).^80^, ^81^ Benefit with the combination therapy was seen in participants from all geographical locations, with comparable safety profiles.^80^, ^81^ The most common side effects/adverse events were hypertension, fatigue and proteinuria in the participants receiving Atezolizumab and Bevacizumab.^80^, ^81^ The risk of major oesophageal variceal haemorrhage is a particular concern to those patients receiving Atezolizumab and Bevacizumab and should be actively monitored for and treated prior to treatment commencing.^80^, ^81^ This has led to regulatory approval by the FDA, European Medicines Agency, and Medicines and Healthcare products Regulatory Agency for the use of Atezolizumab and Bevacizumab in patients with unresectable or metastatic HCC who have not received prior systemic therapy.^43^ Other clinical trials exploring the combination of PD‐L1 inhibitors with anti‐VEGF therapy are currently in progress.^42^, ^43^, ^45^, ^46^, ^47^, ^49^ The RESCUE trial (NCT03463876) is a phase II clinical trial exploring the safety and efficacy of Camrelizumab in combination with Apatinib (a VEGFR‐2 inhibitor) in patients with advanced HCC who have previously received Sorafenib,^83^ and there is also a phase I/II clinical trial (NCT04035876) currently underway in China assessing Camrelizumab and Apatinib as potential neoadjuvant therapy for downstaging/bridging of HCC prior to curative liver transplantation.

## 8 COMBINATION ICI THERAPY

Combination monoclonal antibody therapies targeting some of the most commonly studied immune checkpoints are also currently the subject of interest in advanced clinical trials.^42^, ^43^, ^44^, ^45^, ^46^, ^47^, ^48^, ^49^ One of the cohort arms of the CheckMate 040 trial looked at the safety and tolerability of a combination of Nivolumab and Ipilimumab and found, along with a positive ORR (32%) and duration of response, a manageable safety and toxicity profile which led to regulatory approval by the FDA for use in patients with advanced HCC previously treated with Sorafenib.^46^, ^47^, ^49^, ^84^ This led to the development of the CheckMate 9DW trial (NCT04039607), a multicentre phase III trial comparing the overall survival rates (primary outcome) of a combination of Nivolumab and Ipilimumab against Sorafenib/Lenvatinib in patients with advanced HCC who have not received prior systemic therapy. Moreover, the HIMALAYA trial (NCT03298451) is a multicentre phase III trial exploring the safety and efficacy of Durvalumab (a PD‐L1 inhibitor) both as monotherapy and in combination with Tremelimumab (a CTLA‐4 inhibitor) versus Sorafenib in patients with advanced HCC who have not received prior systemic therapy and are also not eligible for locoregional therapy.^85^

## 9 ICIs IN COMBINATION WITH LOCOREGIONAL THERAPIES

Whereas some clinical trials have sought to combine the use of novel immunotherapy agents, there are also several clinical trials that are combining the use of novel ICIs with previously established locoregional therapies used in the treatment of advanced HCC (as discussed in Section 1).^42^, ^44^, ^45^, ^46^, ^47^, ^48^, ^49^ One of the rationales behind this is their likely complementary mechanisms of actions providing both additive and synergistic effects through the direct actions of locoregional therapies on tumour cells and potentiation of these effects through further stimulation of the immune system with the use of ICIs.^86^, ^87^, ^88^, ^89^, ^90^ Ablation techniques, radiotherapy methods and Doxorubicin (the most commonly used chemotherapy agent in TACE) have previously been shown to induce immunogenic cell death by apoptosis. This stimulates recruitment of DCs to the tumour site and upregulates the expression and antigenic presentation of TAAs, including PD‐1/PD‐L1, following their widespread release via apoptotic mechanisms.^86^, ^87^, ^88^, ^89^, ^90^ Locoregional therapies also induce proinflammatory cytokine release, activation and expansion of other innate and adaptive immune cells including NK cells and cytotoxic T‐cells, and downregulation in the activity of immune suppressive cells including Tregs and MDSCs.^86^, ^87^, ^88^, ^89^, ^90^ Ongoing clinical trials involving combination locoregional and ICI therapies are listed in Table 1.

## 10 ICIs IN COMBINATION WITH SYSTEMIC THERAPIES

Multiple clinical trials are currently being undertaken evaluating the impact of ICIs in combination with multi‐targeted tyrosine kinase inhibitors, as potential treatments for advanced HCC. The LEAP‐002 trial (NCT03713593) is a multicentre, double‐blind, randomised phase III trial evaluating the safety and efficacy of Pembrolizumab and Lenvatinib against Lenvatinib as first‐line therapy for patients with advanced HCC, with OS and PFS being the primary endpoints.^91^ The COSMIC‐312 trial (NCT03755791) is a multicentre, randomised phase III trial looking at a combination of Atezolizumab and Cabozantinib (a multi‐target tyrosine kinase inhibitor), along with an arm containing Cabozantinib as monotherapy, against Sorafenib in order to evaluate the safety and efficacy and their effects on OS and PFS (primary endpoints) in patients with advanced HCC who have not received prior systemic therapy.^92^, ^93^ There was also a recent phase Ib trial (NCT03418922) looking at the safety and tolerability of Nivolumab and Lenvatinib in patients with unresectable HCC. The study found a manageable level of adverse events with an overall ORR of 23%.^94^ This therapy combination has progressed onto a current ongoing phase II trial (NCT03841201) for advanced hepatocellular carcinoma.^95^

## 11 CLINICAL AND PRECLINICAL CELLULAR THERAPIES

### 11.1 Adoptive cell transfer

**FIGURE 4:**
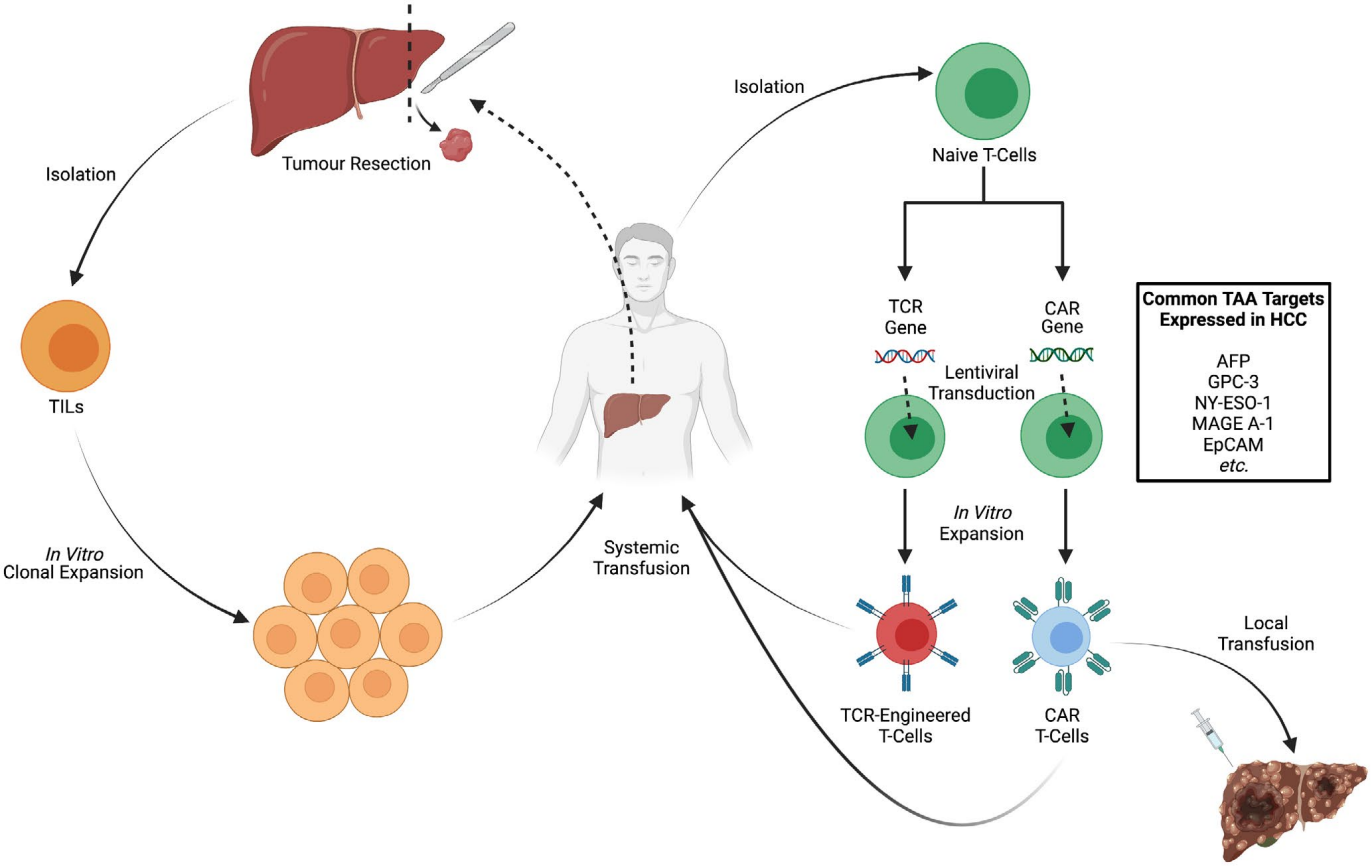
Approaches to adoptive cell therapy, including chimeric antigen receptor (CAR) T‐cells. Earlier approaches relied on isolation of endogenous TILs, whereas later approaches rely on inserting genes encoding receptors (T‐cell receptor [TCR] or CAR) using lentiviral transduction techniques into isolated naïve T‐cells. Following in vitro expansion, adoptive cells are then autologously reintroduced into patients either systemically or locally directly into the tumour site.

Another method in which an individual's immune system can be harnessed to selectively target and kill tumour cells is through ex‐vivo stimulation of T‐cells against TAAs.^102^, ^103^, ^104^ Adoptive cell transfer (ACT) involves identification of TAAs expressed by a tumour cell and priming and expansion of autologous naïve T‐cells against these specific TAAs, prior to reintroduction into the patient (Figure 4).^105^ In the context of HCC, the majority of ACT strategies centre around CD8^+^ cytotoxic T‐lymphocytes, which are capable of direct antitumour effects.^106^, ^107^, ^108^ The most widely studied TAAs expressed by HCC tumour cells are AFP and glypican‐3, although other TAAs are also being studied, including New York esophageal squamous cell carcinoma 1 (NY‐ESO‐1) and melanoma‐associated antigen 1 (MAGE‐A1).^106^, ^107^

Early iterations of ACT therapies include TILs and T‐cell receptor (TCR)‐engineered T‐cells. These broadly comprise of endogenously or in vitro‐isolated T‐cells that target specific TAAs which are expanded in vitro before being transfused back into a host to generate a systemic immune response against tumour cells (Figure 4).^102^, ^103^, ^104^, ^105^ A phase I clinical trial exploring the safety of TILs in 15 patients, who had previously undergone hepatic resection of HCC, demonstrated successful expansion of TILs in 88% of the study population, with an adequate safety profile.^109^ However, issues surrounding the efficacy and feasibility of delivering such personalised therapies to patients remain.^102^, ^103^, ^104^, ^105^

A more recent approach to ACT therapy involves engineering of chimeric antigen receptors (CARs), comprising of an extracellular single‐chain variable fragment that functions as the TAA‐recognition domain along with costimulatory molecules, a transmembrane domain and intracellular signalling domains, on T‐cells (Figure 4).^110^, ^111^, ^112^ These cells provide a more sophisticated approach compared to traditional ACT models given the greater ability to control most, if not all, aspects of T‐cell function. These include recognition of a specific TAA by the chimeric TCR, to activation of specific downstream cell signalling pathways, and generation of a targeted immune response.^110^, ^111^, ^112^ CAR T‐cells are also able to circumvent any previous requirements of the TAA being loaded onto an HLA complex in order to achieve T‐cell activation (this process often being downregulated by tumour cells as part of their immune evasion strategy).^110^, ^111^, ^112^ Great strides have been made in the development of CAR T‐cells used to treat cancer and this has been reflected in the recent regulatory approval by the FDA of two different CAR T‐cell therapies for the treatment of acute lymphoblastic leukaemia—Tisagenlecleucel/Kymriah—and diffuse large B‐cell lymphoma—axicabtagene ciloleucel/Yescarta—respectively.^110^, ^111^, ^112^

There are currently multiple ongoing phase I/II clinical trials examining the safety profile and tolerability of CAR T‐cells in HCC. They broadly target some of the most commonly expressed TAAs on HCC tumour cells including GPC‐3, AFP, and NY‐ESO‐1.^106^, ^107^, ^108^ There is also an ongoing phase I/II trial (NCT03013712) currently exploring the safety and efficacy of CAR T‐cells that target the epithelial cell adhesion molecule antigen expressed on a variety of tumour cells including HCC.

One of the main issues surrounding CAR T‐cell therapies are the well‐characterised but serious adverse effects, including cytokine release syndrome and neurotoxicity, particularly when CAR T‐cells are delivered to individuals systemically.^110^, ^111^, ^112^ Moreover, specific concerns surrounding the efficacy of systemic CAR T‐cell therapy in HCC exist, including the unfavourable tumour macroenvironment, characterised by fibrotic stromal changes and cirrhosis along with a reduced blood supply, thereby limiting penetration by systemic CAR T‐cells.^106^ In an attempt to reduce the risk of such unwanted side effects and improve the overall efficacy of CAR T‐cells, some recent efforts have focused on the feasibility and safety of more localised delivery of CAR T‐cells directly to the site of the tumour, primarily through locoregional methods.^106^

### 11.2 Dendritic cell therapies

The concept of developing a so‐called anti‐cancer vaccine that primes and stimulates the adaptive immune system to target and destroy tumour cells has existed for over 60 years and is a method that has gained particular interest and traction over the past 20 years. The first anti‐cancer vaccine—sipuleucel‐T/Provenge—secured regulatory approval by the FDA for the treatment of metastatic castration‐resistant prostate cancer in 2010.^113^, ^114^, ^115^, ^116^ This strategy broadly involves isolating autologous peripheral blood mononuclear cells in vitro, expanding the population of DCs through the addition of co‐stimulating factors including granulocyte‐macrophage colony‐stimulating factor (GM‐CSF) and IL‐4, before incubating the mature DCs with autologous tumour lysate or with specific TAAs.^113^, ^114^, ^115^, ^116^ In the case of sipuleucel‐T/Provenge, DCs are incubated with recombinant prostate acid phosphatase.^113^, ^114^, ^115^, ^116^ This procedure generates fully primed APCs that are able to present antigenic peptides via the major histocompatibility complex on their cell surface.^113^, ^114^, ^115^, ^116^ These can then be infused back into a host to stimulate an adaptive cell‐mediated immune response, characterised by activation and clonal expansion of a variety of host cells including CTLs that specifically target and kill tumour cells.^117^

It has previously been demonstrated that the surrounding tumour microenvironment in HCC is highly immunosuppressive.^42^, ^44^, ^49^ One of the aspects of this relative state of immunosuppression is due to an overall reduction in the number of DCs found locally within the liver.^49^ Thus, DC therapy represents a theoretical niche whereby supplementing the immune system with primed autologous DCs could potentially tip the balance of the immune system in favour of the host and promote an overall antitumour immune response.^113^, ^115^, ^116^

Early‐phase clinical trials involving DC vaccines have shown them to be generally safe and well‐tolerated when targeting TAAs expressed on HCC tumour cells, both as a primary therapy and as an adjunctive therapy in combination with other established treatments, along with being associated with improved overall efficacy and survival outcomes.^118^, ^119^ However, it remains unclear as to which DC incubation strategy is the most effective for treating HCC. Earlier clinical trials focused on incubating autologous DCs with whole tumour cell line lysates derived from the HepG2 cell line, with a level of success in terms of safety/tolerability and effects on mOS.^120^, ^121^ Controversies exist regarding the most appropriate administration route for DC therapy (similar as with CAR T‐cells) and the most effective therapeutic combinations (e.g. surgical resection/curative treatment, locoregional therapies, other systemic and/or immunotherapies, etc.).^122^ There have also been multiple clinical trials undertaken using a single or a small combination of well‐established TAAs in HCC, including AFP, GPC‐3, MAGE‐1, etc., with mixed results.^74^, ^107^, ^108^, ^122^ This could be due to a lack of adjuvant signalling or tumour escape in response to single‐antigen vaccination. This has led to the development of the HEPAVAC project (www.hepavac.eu), a study looking to identify further novel TAAs specific to HCC. The HepaVac‐101 trial (NCT03203005), a phase I/II clinical trial is exploring the safety, tolerability and immunogenicity of an adjuvant therapeutic anti‐cancer vaccine targeting 16 recently identified novel peptides from resected HCCs (IMA970A) in combination with a novel co‐stimulatory agent (CV8102) in patients with early‐to‐intermediate‐stage HCC following the failure of standard treatment.^123^

## 12 THERAPEUTIC VIRUSES

Viruses are obligate intracellular pathogens that utilise unique proteins in their machinery to undertake viral replication in target host cells before escaping generally by cellular lysis (thereby inducing cell death) in order to continue their spread. These principles, in part, have led to the creation and evolution of oncolytic viruses (OVs), which broadly exert their antitumour effects through multiple mechanisms.^124^, ^125^, ^126^, ^127^ Firstly, OVs often replicate preferentially in malignant cells and can be additionally genetically engineered to target specific host tumour cells. This is achieved either through recognition of and binding to TAA cell‐surface markers on tumour cells, or by incorporating or deleting various genes from the viral genome that promote preferential survival and viral replication in targeted tumour cells.^124^, ^125^, ^126^, ^127^ Secondly, OVs promote immunogenicity within the host immune system via multiple mechanisms, including the induction of interferons, the release and uptake of TAAs by APCs or by being genetically engineered to express genes that upregulate both humoral and cell‐mediated immune responses.^124^, ^125^, ^126^, ^127^ The field of OVs has previously found success following the regulatory approval by the FDA of a modified herpes simplex virus type 1, talimogene laherparepvec, which was shown to improve both the rates of objective overall response to treatment and mOS in patients with advanced melanoma in a phase III clinical trial.^128^

The most commonly studied OVs for the potential treatment of HCC in both preclinical and clinical studies include adenovirus and vesicular stomatitis virus (VSV), both due to their ability to preferentially infect HCC tumour cells, along with herpes simplex virus, Edmonston strain of the measles virus, Newcastle disease virus, and vaccinia virus.^108^, ^129^, ^130^ One of the leading OVs targeting HCC currently being investigated in clinical trials is a modified Wyeth strain of the vaccinia virus, JX‐594/pexastigmogene devacirepvec (Pexa‐Vec), which has been genetically engineered to express GM‐CSF in order to promote host antitumour activity in tumour cells typically expressing high levels of thymidine kinase following deletion of the thymidine kinase gene from its viral genome.^108^, ^129^, ^131^ Unfortunately, the TRAVERSE trial (NCT01387555), a phase IIb trial comparing intravenous and intratumoural injections of Pexa‐Vec to placebo as second‐line therapy in patients with advanced HCC who had previously failed Sorafenib therapy, did not meet its primary endpoint of OS.^132^ Moreover, the PHOCUS trial (NCT02562755), a phase III trial comparing Pexa‐Vec with Sorafenib as first‐line to Sorafenib monotherapy in patients with advanced HCC,^133^ was terminated early following the failure of a planned interim futility analysis indicating that the study was unlikely to meet its OS primary outcome.^108^ Despite these setbacks, other clinical trials involving different OVs, including H101 recombinant human adenovirus type 5 and VSV expressing human interferon beta, are currently ongoing.^129^

Promotion of an immunogenic environment by OVs (as discussed above) also further lends itself to complementing the immunogenic activity of ICIs which has the potential to provide an increased and/or synergistic antitumour response.^134^, ^135^ There are currently several clinical trials underway exploring the efficacy of OVs and ICIs in combination for the treatment of multiple solid tumour sites.^134^, ^135^ In the context of HCC, OVs hold potential to sensitise the immune‐tolerant microenvironment and to immunologically prime tumours for ICI activity.^131^, ^136^

## 13 CONCLUSION

Until recently, there were very few successes in the development of systemic therapies for advanced HCC. However, rapid advances in the field of cancer immunotherapy have allowed new targeted therapies to be developed. For a long period of time, aside from Sorafenib (and more recently Lenvatinib), there remained no other first‐line treatments licensed and available for the systemic treatment of advanced HCC. This was until the recent success of the IMbrave150 clinical trial which demonstrated a clinical benefit for Atezolizumab/Bevacizumab combination therapy. Other successes within this field include the licensing and regulatory approval by the FDA of both Pembrolizumab and Nivolumab/Ipilimumab combination as second‐line therapies for the treatment of advanced HCC. Multiple clinical trials in various stages are currently in the pipeline exploring the potential use of ICIs both as monotherapies and in combination with other established treatment modalities for the treatment of advanced HCC.

Whilst ICI immunotherapy leads the field in the development of new systemic therapies for treating advanced HCC, other immunotherapeutic modalities are rapidly progressing, including CAR T‐cells, DC vaccines and OVs. Much of this work has been aided by a better knowledge and understanding of the commonly expressed TAAs on HCC tumour cells and of the interplay between tumour cells and their surrounding microenvironment. Technological advances have also led to the development of the next generation of monoclonal antibodies and CAR T‐cells, improved personalisation of DC vaccines, and better genetic engineering of OVs. These more sophisticated therapies are being clinically tested to enable more specific targeting of tumour cells, better control of downstream immune responses and a reduction in off‐target effects. Moreover, several clinical trials are currently underway exploring the role of immunotherapies in the neoadjuvant and adjuvant settings in patients with early‐ and intermediate‐stage HCC, in an effort to improve treatment outcomes and prevent tumour recurrence. Outcomes from future clinical trials should also aim to investigate a similar role for immunotherapies as downstaging therapy in advanced HCC in order to potentially widen access to other treatments used for early and immediate‐stage HCC in these individuals. Future research in this field should consider the potential risks of treatment hepatotoxicity at the start of the research pipeline. Finally, determining clinically useful biomarkers as surrogate measures of immunotherapy treatment response and prognosis in HCC remains the focus of many in vitro and in vivo studies.

## AUTHOR CONTRIBUTION

Justin K. H. Liu and Adel Samson were involved in the initial conception and design of the manuscript. All of the authors contributed towards drafting the manuscript and approved the final version.

## CONFLICT OF INTEREST

The authors declare no conflict of interest.

## ETHICAL APPROVAL STATEMENT

Ethical approval was not required for this study.

## Data Availability

Data sharing is not applicable to this article as no new data were created or analysed in this study.
